# Highly Efficient and Reversible Covalent Patterning of Graphene: 2D‐Management of Chemical Information

**DOI:** 10.1002/anie.201914088

**Published:** 2020-01-29

**Authors:** Tao Wei, Malte Kohring, Muqing Chen, Shangfeng Yang, Heiko B. Weber, Frank Hauke, Andreas Hirsch

**Affiliations:** ^1^ Department of Chemistry and Pharmacy & Joint Institute of Advance Materials and Process (ZMP) Friedrich-Alexander University of Erlangen-Nürnberg Nikolaus-Fiebiger-Strasse 10 91058 Erlangen Germany; ^2^ Department of Applied Physics & Institute of Condensed Matter Physics Friedrich-Alexander University of Erlangen-Nürnberg Staudtstrsse 7/ Bau A3 91058 Erlangen Germany; ^3^ Department of Materials Science and Engineering University of Science and Technology of China Hefei 230026 China

**Keywords:** covalent patterning, graphene, patterned functionalization, Raman spectroscopy

## Abstract

Patterned graphene‐functionalization with a tunable degree of functionalization can tailor the properties of graphene. Here, we present a new reductive functionalization approach combined with lithography rendering patterned graphene‐functionalization easily accessible. Two types of covalent patterning of graphene were prepared and their structures were unambiguously characterized by statistical Raman spectroscopy together with scanning electron microscopy/energy‐dispersive X‐ray spectroscopy (SEM‐EDS). The reversible defunctionalization processes, as revealed by temperature‐dependent Raman spectroscopy, enable the possibility to accurately modulate the degree of functionalization by annealing. This allows for the management of chemical information through complete write/store/erase cycles. Based on our strategy, controllable and efficient patterning graphene‐functionalization is no longer a challenge and facilitates the development of graphene‐based devices.

## Graphical abstract

**Writing on graphene**: Patterned graphene functionalization with a tunable degree of functionalization was achieved by using a reductive protocol combined with lithography. The structures were unambiguously confirmed by statistical Raman spectroscopy and SEM‐EDS. Based on this strategy, controllable and patterned graphene functionalization is no longer a challenge, which facilitates the development of graphene‐based devices.

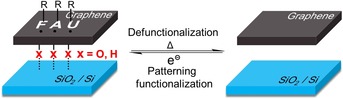

Nanoengineering of monolayer graphene by surface patterning remains a highly attractive concept for the rational design of well‐defined 2D materials.^1^, ^2^, ^3^, ^4^ This goes along with the possible realization of unprecedented combinations of functions generated by the spatial arrangement of patterned surface regions in confined space comprising, for example, controlled wetability, catalytic activity, molecular recognition, optical activity, electron‐ or energy‐transfer capability, and electron conductivity. The possibilities are numerous and very exciting. To fully exploit these opportunites, efficient and flexible methods for 2D patterning have to be elaborated. In recent years, a couple of first promising approaches towards this goal have been reported. So far, two main strategies can be distinguished: a) Carving out graphene nanostructures (for example, ribbons) by etching away defined parts of the graphene lattice^5^, ^6^, ^7^, ^8^ and b) patterning by covalent binding of addends at defined lattice regions.^9^, ^10^, ^11^, ^12^, ^13^ The latter approach allows for the possibilty of introducing the above‐mentioned surface functionalities, which are located next to the electrically conductive regions of intact graphene. Further approaches include patterning via N‐atom incorporation into the C‐lattice (formation of spatially resolved heterographene regions)^14^ and patterned dehydrogenation of hydrogenated graphene.^15^ So far, covalent patterning with organic addends such as diazonium salts^9^, ^10^ and, in particular, Diels–Alder‐additions^13^ provided only very low degrees of additions, which is reflected by low *I*_D_ */I*_G_ values (Raman D‐ and G‐peak intensities; in most cases much lower than 1). Covalent pattering by fluorination can provide considerably higher degrees of addition.^16^ However, the drawback of this approach is that the σ‐lattice can also be attacked, and thermal treatment leads to destruction rather than to restauration of the intact graphene framework after addend cleavage. In general, such chemical procedures have been combined with classical patterning techniques such as lithography and direct laser (or plasma) writing.^17^, ^18^, ^19^, ^20^, ^21^ In this way, a first series of 2D‐patterened graphene samples has been synthesized.^5^, ^6^, ^7^, ^8^, ^9^, ^10^, ^11^, ^12^, ^13^, ^14^, ^15^, ^16^ For instance, by using Diels–Alder chemistry in combination with lithography, Wan et al. synthesized functional patterned graphenes which exhibit improved conductivity and can be used as transparent electrodes.^13^ Following a similar strategy, functional cyclopentadienes were attached by Braunschweig and co‐workers.^12^ On the basis of lithography, hydrogenation, and subsequent diazonium chemistry, Tour realized the formation of graphene/hydrographene superlattices, which were investigated with respect to their sensor behaviour.^10^ However, as pointed out above, the degrees of organic‐addend binding of these covalent approaches are still comparatively low (*I*_D_/*I*_G_=0.25–0.80).

We now present a facile and efficient method for patterned graphene‐functionalization, which provides comparatively high covalent‐addition degrees. At the same time, this covalent graphene‐functionalization is completely reversible. This allows for the management of chemical information by establishment of complete write/store/erase cycles. Our approach combines graphene‐reactivity principles which we recently discovered with classical manufacturing methods (Figure 1). These are: a) Highly efficient covalent addend binding by reductive activation of graphene with alkali metals;^22^ b) enabling covalent addend binding by switching on strain‐free antaratopic additions provided by the reactive underlying substrate (SiO_2_/Si),^[23.24]^ and c) spatial structuring using electron‐beam lithography (EBL) on a polymethyl methacrylate (PMMA) thin film.

**Figure 1:**
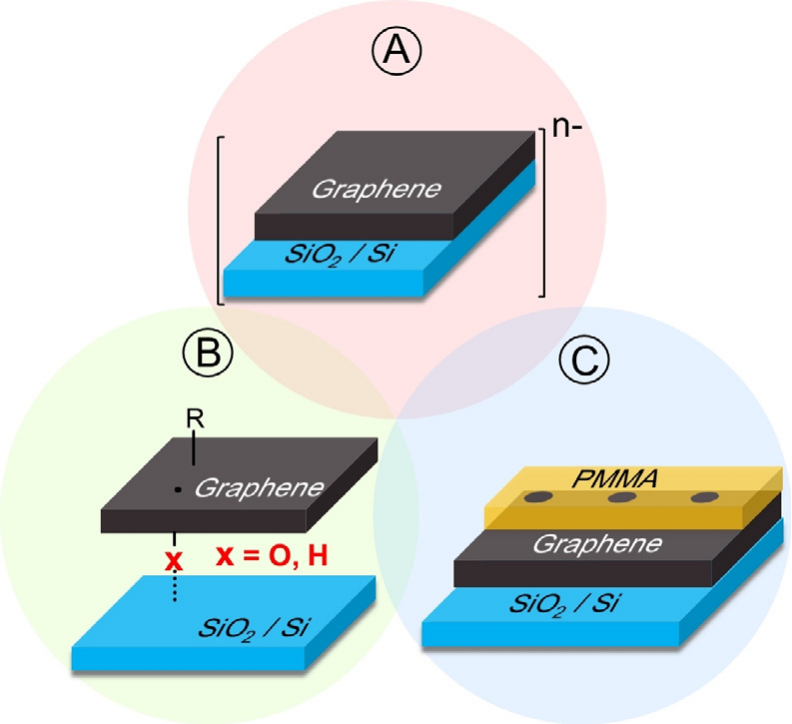
A) Combined principles for efficient covalent 2D patterning of graphene. Activation by negative charging. B) Enabling of strain‐free antaratopic additions provided by surface atoms (O, H) from the underlying SiO_2_/Si substrate. Inert substrates would allow only for successive supratopic additions, which, however, are forbidden due the enormous amout of strain energy that would be built up.^23^ C) EBL‐patterned PMMA coverage leaving spatially defined regions of reduced graphene accessible for covalent addend binding.

The overall procedure introduced here allows for straightforward 2D patterning of graphene with organic addends. The degrees of covalent organic functionalization are considerably higher (up to more than one order of magnitude) than those reported before (Table S3, Supporting Information). Subsequent thermal treatment of our 2D‐structured organo‐graphenes allows for the controlled down‐regulation of the extend of addend binding even down to complete defunctionalization and restauration of the entire sp^2^‐lattice.

Our reductive 2D‐patterning sequence of graphene monolayers deposited on a SiO_2_/Si substrate (Scheme 1) started with the preparation of a polymethylmethacrylat (PMMA) mask. For this purpose, we used an adapted polymer‐film‐deposition and lithography procedure^7^ generating two different periodic patterns (cyclic dots or FAU) via electron‐beam lithography (for details, see the Supporting Information, Sections S1 and S2). This allowed us to selectively expose uncovered graphene regions to subsquent addend‐binding reactions. The reductive activation of the unprotected graphene regions was accomplished by treatment with a Na/K alloy. Once the Na/K alloy was blown off with Ar, subsequent addend binding was achieved by exposure with the arylating electrophiles nitro‐ or bromo‐benzenediazonium tetrafluoroborate dissolved in ethanol, which is not a solvent for PMMA. After removal of excess reagent by washing with ethanol and water, the PMMA layer was removed by disolution in acetone. The entire procudure provided efficient 2D graphene patterning exemplified by the sheet architectures G_A_ and G_B_ (Scheme 1).

**Scheme 1:**
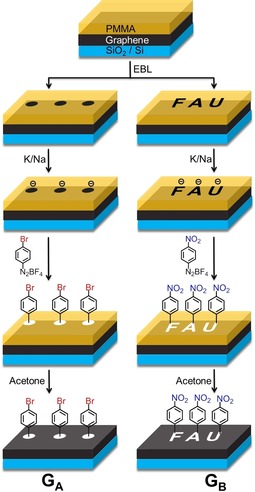
Schematic illustration of the reaction sequence for the patterned functionalization of monolayer graphene. The dark cyclic dots and the FAU logo are the exposed (unprotected) graphene, while the other areas of (protected) graphene are still covered by the orange PMMA. The PMMA coverage can be removed by treatment with acetone, leaving the addend‐structured sheet G_A_ and G_B_ (the addend‐carrying regions are indicated in white). The diameter of a dot is 5 μm, the length and width of the FAU logo is 20 μm and 30 μm, respectively.

In Figure 2 A, the mean Raman spectra of both the protected and unprotected regions after the chemical reaction leading to the 2D sheet G_A_ are displayed. Clearly, before functionalization, the patterned region within G_A_ exhibits a pronounced G‐band at ≈1582 cm^−1^ assigned to a sp^2^ carbon lattice, while the defect‐induced D‐band located at ≈1350 cm^−1^ is indiscernible. The ratio of the intensity of the d‐band to the G‐band (*I*_D_/*I*_G_) was calculated to be <0.1. This result excludes the formation of radiation‐related defects introduced within the patterned region of G_A_ during the process of electron‐beam lithography. In the mean Raman spectrum of the patterned region of G_A_ after functionalization, a significantly more intense D‐band appears, indicating the conversion of sp^2^–sp^3^ basal carbon atoms as a result of covalent functionalization, leading to a considerably higher *I*_D_/*I*_G_ ratio of about 2.6. Since the *I*_D_/*I*_G_ ratio is known as a measure for the degree of functionalization, an efficient covalent functionalization for the patterned region within G_A_ is demonstrated. This observed degree of functionalization is significantly higher than that of previously reported cases of graphene patterning,^9^, ^10^, ^11^, ^12^, ^13^, ^14^, ^15^, ^16^ emphasizing the superiority of our functionalization protocol. Interestingly, after reaction, the PMMA‐protected regions do not exhibit any defect‐induced D peaks in their Raman spectra, indicating that the covered graphene remains intact. As a conseqeunce, our reductive graphene‐functionalization concept, selectively taking place in the uncovered regions, provides a very simple and efficient method for 2D graphene patterning, with an unprecedented high degree of sp^2^‐to‐sp^3^ conversion. To visualize a typical high‐resolution pattern (micrometer scale), large‐scale Raman mapping (in an area of 70×80 μm^2^) was carried out and the corresponding pattern image (series of circles of 5 μm diameter) can be clearly distinguished through *I*_D_/*I*_G_ Raman mapping (Figure 3 B). Furthermore, it can be clearly seen that the corresponding *I*_D_/*I*_G_ ratio of almost all these patterned regions exhibits a uniform distribution with a very high *I*_D_/*I*_G_ ratio of 2.6 (Figures 3 B and 4 A).

**Figure 2:**
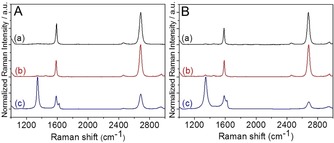
Raman analysis of the 2D patterning of monlayer graphene. a) Unprotected region of G_A_ (A) and G_B_ (B) before functionalization; b) PMMA‐protected region of G_A_ (A) and G_B_ (B) after polymer removal; c) Unprotected region of G_A_ (A) and G_B_ (B) after functionalization. Averaged spectra (≈200 single‐point spectra) of the respective samples, *λ*_exc_=532 nm.

**Figure 3:**
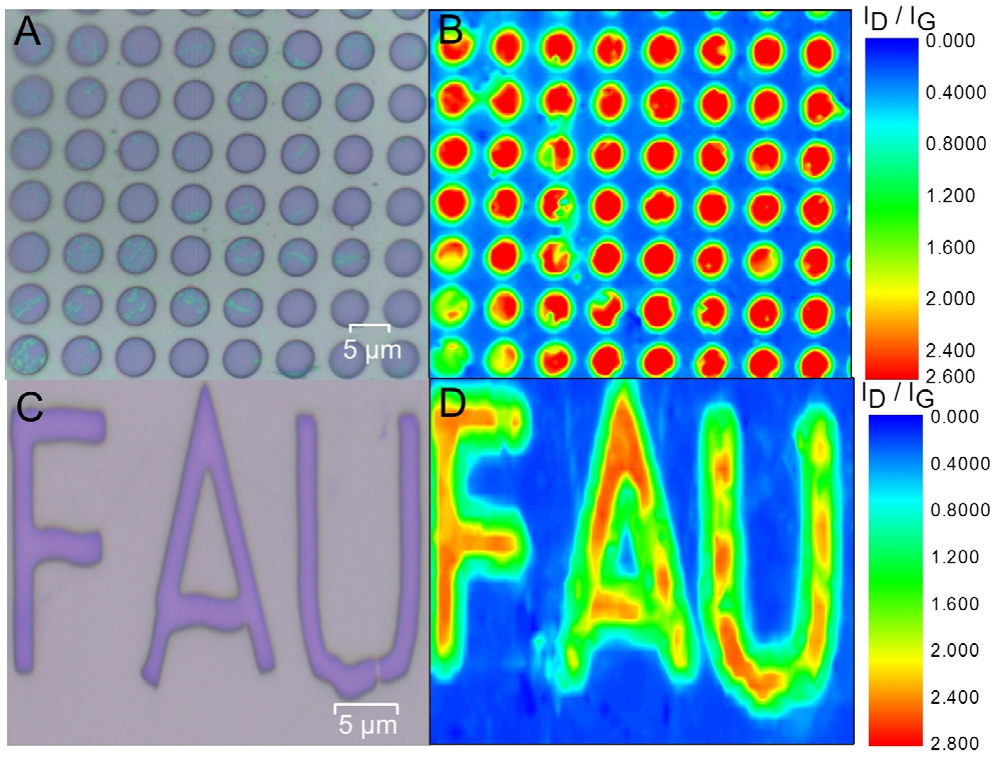
A), C) Optical images of PMMA‐patterned graphene (precursor masks) for G_A_ (A) and G_B_ (C). The blue regions represent exposed graphene. B), D) Corresponding Raman *I*_D_/*I*_G_ mapping images of G_A_ (B) and G_B_ (D) after addend binding and removal of the PMMA layer.

**Figure 4:**
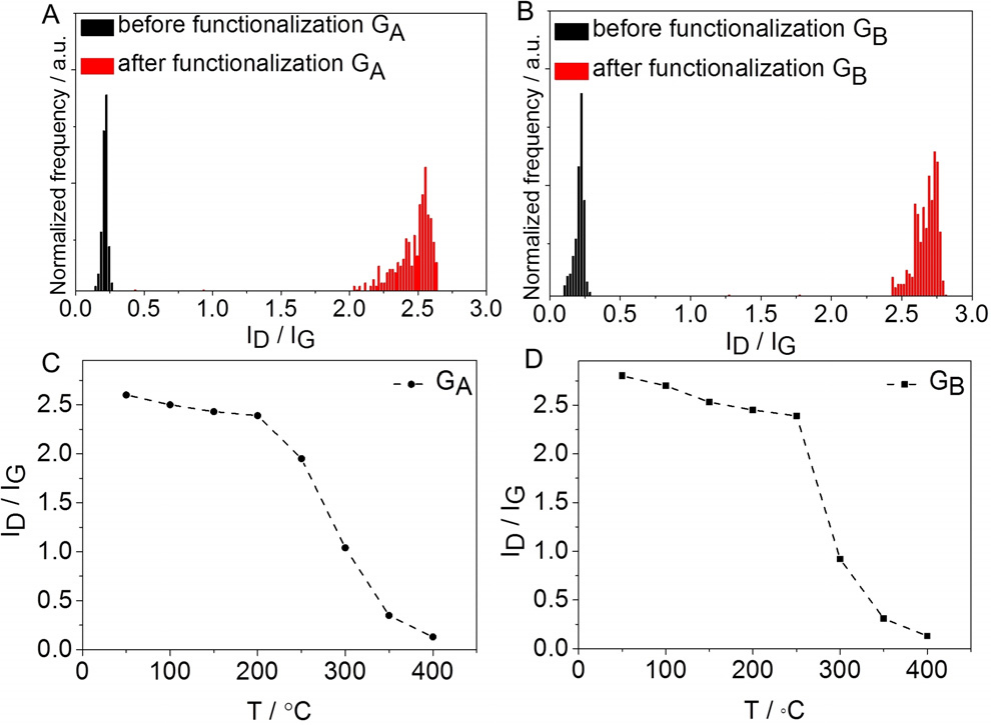
Statistical Raman histograms and mean Raman *I*_D_/*I*_G_ ratio extracted from temperature‐dependent Raman spectra of G_A_ (A,C) and G_B_ (B,D).

In the case of G_B_, the same results, that is, a dramatically increased *I*_D_/*I*_G_ ratio only for the patterned region was revealed, and the PMMA‐protected regions remain unchanged. This increased *I*_D_/*I*_G_ ratio of about 2.8 (Figures 3 D and 4 B) resembling that of G_A_ indicates also very efficient functionalization due to the reductive activation. The large‐scale Raman *I*_D_/*I*_G_ mapping (area of 30×50 μm^2^) showing a clear pattern image (FAU logo) strongly supports the successfully patterned functionalization for G_B_ as well (Figure 3 D).

Additionally, temperature‐dependent Raman spectroscopy measurements were carried out under an inert‐gas atmosphere to investigate thermally induced bond cleavage on G_A_ and G_B_. It can be seen that, as the temperature rises, the intensity of the D‐bands of both G_A_ and G_B_ decreases down to the value of intact graphene (*I*_D_/*I*_G_=0.08) at about 400 °C (Figures 4 and 5). This clearly demonstrates the complete restauration of the sp^2^ carbon network of the graphene basal plane by the thermal cleavage of the covalently bound aryl addends. The main mass‐loss step (covalent bond cleavage) sets in at 200–250 °C and extends to around 400 °C. This completely reversible 2D patterning of graphene due to reductive functionalization and thermal defunctionalization stands out since it combines, for the first time, two advantages (Scheme 2): a) facile and efficient functionalization (write/store) b) complete reversibility and recovery of intact graphene (erase). Only graphene fluorination can provide similar high degrees of functionalization.^16^, ^25^ However, in this case, thermal treatment leads to the complete decomposition of the carbon framework rather than clean conversion to graphene.^26^ This excellent reversibility of our approach opens the opportunity of varying both the nature of the addend and the degree of functionalization in a wide range through a precise adjustment of the annealing temperature. Since the chemical functionalization of graphene will exert a great impact on its electronic properties, our ability to control graphene chemistry shown here could be an important step to manipulate the electronic band gap of graphene to obtain a rare, continuously tunable band gap (in view of its high reversibility). As a consequence, it may help this 2D‐patterning architecture to find extensive applications in semiconducting (for example, chemical sensors) or insulating nanodomains.

**Figure 5:**
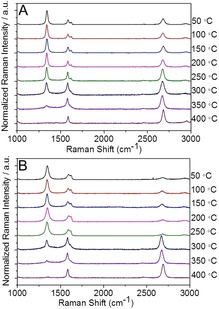
Temperature‐dependent statistic Raman spectra of G_A_ (A) and G_B_ (B), *λ*_exc_=532 nm.

**Scheme 2:**
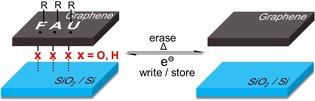
Reductive patterning functionalization and complete defunctionalization of monolayer graphene on a SiO_2_/Si substrate.

As pointed out above, the defunctionalization process for both G_A_ and G_B_ occurs predominantly in the temperature region between 250–400 °C. Thus, for example, by annealing at 250 °C, we can adjust the *I*_D_/*I*_G_ ratios of these two patterned functionalized graphenes G_A_ and G_B_ to around 2 (Figure 5 A,B). To get a good impression, the large‐scale Raman mapping of annealed G_A_ very clearly shows the distinct pattern with a decreased *I*_D_/*I*_G_ ratio of around 2 encoded in predominantly green color (Figure 6 A). The *I*_D_/*I*_G_ ratio can be further decreased by increasing the annealing temperature to 300 °C, as clearly demonstrated by the large‐scale Raman mapping of G_B_, where the distinct pattern image with an *I*_D_/*I*_G_ ratio of around 1 is encoded in turquoise color (Figure 6 C). Annealing at an even higher temperature of 400 °C resulted in the complete defunctionalization of both G_A_ and G_B_, as revealed by the large‐scale Raman mapping (Figure 6 B,D). The local degree of functionalization of both G_A_ and G_B_ adjusted with different annealing temperatures has been quantified (Table S2). Altogether, the statistical Raman spectroscopy combined with temperature‐dependent Raman spectroscopy explicitly confirms that the patterned functionalization of graphene with a controllable degree of functionalization is feasible by our proposed protocol.

**Figure 6:**
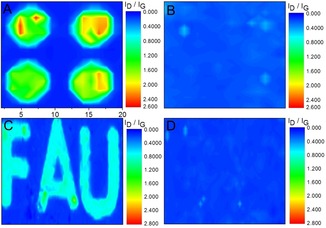
Raman *I*_D_/*I*_G_ mapping image of G_A_ after A) 250 °C and B) 400 °C annealing, and of G_B_ after C) 300 °C and D) 400 °C annealing, *λ*_exc_=532 nm.

For further characterization of the chemical nature of 2D‐patterened G_A_ and G_B_, we carried out scanning electron microscopy/energy‐dispersive X‐ray spectroscopy (SEM‐EDS) measurements in order to probe the element distributions (Figure 7). For the preparation of G_A_ and G_B_, two different electrophiles, that is, 4‐bromobenzene diazonium tetrafluoroborate and 4‐nitrobenzene diazonium tetrafluoroborate, respectively, were used. Thus, it is conceptually reasonable to assume that the elements Br and N will be exclusively present in the patterned regions of G_A_ and G_B_. As expected, the SEM‐EDS measurement of the Br distribution of G_A_ shows a clear pattern‐related distribution. A similar element distribution exclusively present in the patterned region was also found in G_B_ for nitrogen. These results nicely corroborate the successful 2D patterning of graphene in G_A_ and G_B_, in good accordance with the Raman analysis. Moreover, the elements Br and N are distributed over the entire patterned region within G_A_ and G_B_, once again suggesting very homogeneous functionalizations. Significantly, no element signals outside of the patterned regions could be identified, indicating the efficiency of washing away non‐covalently bound species during work‐up. This independently demonstrates the covalent nature of the addend patterning.

**Figure 7:**
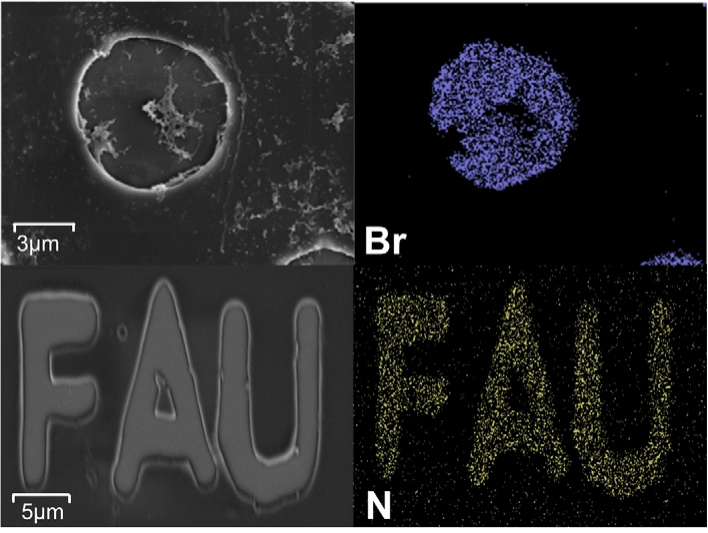
SEM images and EDX elemental maps of G_A_ and G_B_. Element maps of Br‐L (blue) and N‐K (yellow) are shown for G_A_ and G_B_, respectively.

In summary, for the first time, a reductive functionalization protocol using a Na/K alloy in combination with lithography was presented as a facile and efficient route for patterning graphene functionalization. The approach afforded two types of graphene derivatives. G_A_ and G_B_, with a lithographically defined spatial resolution. Their successful patterning functionalizations were unambiguously characterized by statistical Raman spectroscopy and SEM‐EDX. Furthermore, assisted by temperature‐dependent Raman investigations, their complete reversible defunctionalization processes were revealed, which is of great importance for the management of chemical information by establishment of complete write/store/erase cycles. Our work described here dealing with a long‐standing challenge on patterning functionalization of graphene represents a significant advantage in graphene chemistry and will pave the way for this exciting material towards various applications.

## Conflict of interest

The authors declare no conflict of interest.

## Supporting information

As a service to our authors and readers, this journal provides supporting information supplied by the authors. Such materials are peer reviewed and may be re‐organized for online delivery, but are not copy‐edited or typeset. Technical support issues arising from supporting information (other than missing files) should be addressed to the authors.

Supplementary
