# Supplementary material for: Highly Efficient and Reversible Covalent Patterning of Graphene: 2D‐Management of Chemical Information
Source: Angew Chem Int Ed Engl. 2020 Jan 29;59(14):5602–6. doi: 10.1002/anie.201914088 (PMC7154694; doi:10.1002/anie.201914088)
Supplement: Supplementary file 1 — Supplementary [file ANIE-59-5602-s001.pdf]

Angewandte Chemie
Eine Zeitschrift der Gesellschaft Deutscher Chemiker

Supporting Information

Highly Efficient and Reversible Covalent Patterning of Graphene: 2D-Management of Chemical Information

*Tao Wei, Malte Kohring, Muqing Chen, Shangfeng Yang, Heiko B. Weber, Frank Hauke, and Andreas Hirsch**

anie_201914088_sm_miscellaneous_information.pdf

Content

Experimental section.

S1. Patterned graphene fabrication.

S2. Patterned graphene functionalization of G_A and G_B.

S3. Quantified local degree of functionalization of G_A and G_B.

Experimental section.

Materials. CVD graphene on 1×1 cm^2 Polymethylmethacrylate (PMMA) substrate was purchased from ACS material Co (USA). All other chemicals were purchased from Sigma Aldrich Co. (Germany) and used without further treatment. The solvent of ethanol was dried for three days over 4 Å molecular sieves, which were preheated under vacuum for another three days. Subsequently, the dried ethanol was degassed by pump freeze (seven iterative steps) and transfer to glove-box (< 0.1 ppm O_2, < 0.1 ppm H_2O) for using.

Raman Spectroscopy: The Raman spectroscopic characterization was performed on a Horiba Jobin Yvon LabRAM Aramis. A laser (Olympus LMPlanFl50x, NA 0.50) with an excitation wavelength of 532 nm and a spot size of ~ 1 µm was used. The spectrometer was calibrated by using crystalline graphite. Spectral data was obtained through a motorized x-y table in a continuous line scan mode (SWIFT-module).
The temperature dependent Raman measurements were performed in a Linkam stage THMS 600, equipped with a liquid nitrogen pump MS94 for temperature stabilization under a constant flow of nitrogen. The measurements were carried out on Si/SiO_2 wafers with a heating rate of 10 K/min.

Scanning electron microscopy-energy dispersive X-ray spectroscopy: Scanning electron microscopy-energy dispersive X-ray spectroscopy was performed on GeminiSEM 500 equipped with oxford X-max 150. The working conditions were set at an operating at accelerating voltage of 5 kV, working distance of 7.3mm, the elevation angle of detector is 35° and the sample is vertical to the secondary-electron emission.

S1. Patterned graphene fabrication.

The sample was spin-coated with a Polymethylmethacrylate (PMMA) double layer (PMMA 200 k 100 nm, PMMA 950 k 200 nm), followed by a bake step after each layer (Layer 1: 180°C, 60 s; Layer 2: 180°C, 90 s). Subsequently two different patterns (Fig. S1 and S2) in the double layer was created by performing e-beam lithography with a Zeiss Supra SEM (10kV). Such conditions avoid any radiation-related defects in the graphene layer[1]. Irradiated PMMA areas were removed by isopropanol-methyl isobutyl ketone solution.

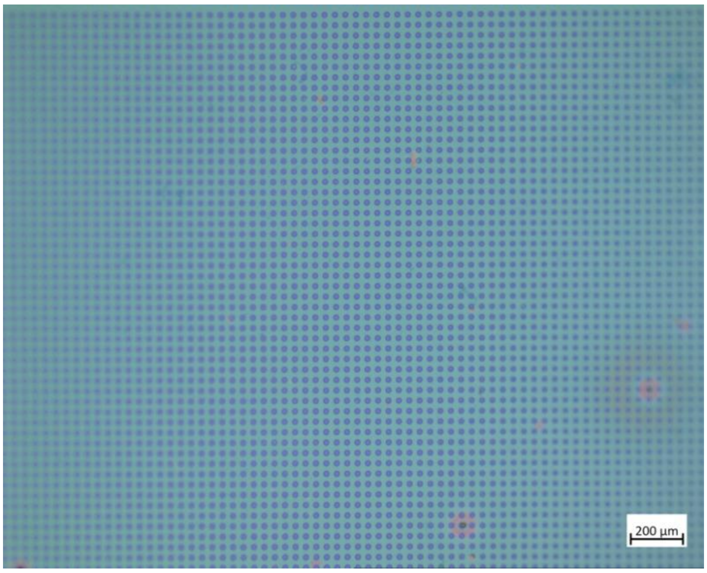

Figure S1. Patterned graphene within G_A. The dark blue cyclic dots are areas where PMMA is removed such that the graphene is exposed.

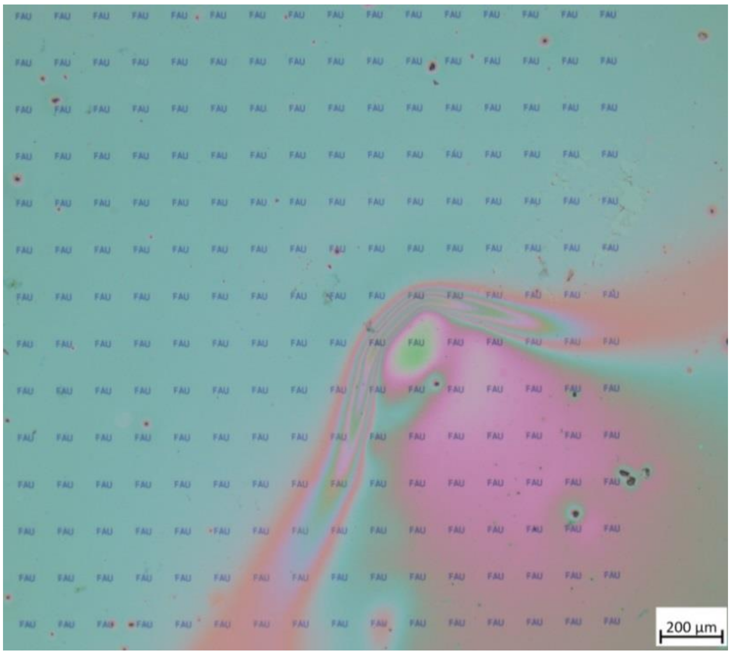

Figure S2. Patterned graphene within G_B. The dark blue FAU logos are areas where the PMMA is removed such that

the graphene is exposed.

S2. Patterned graphene functionalization of G_A and G_B.

Inside the glovebox (< 0.1 ppm O, < 0.1 ppm H_2O, Ar), the above prepared patterned graphene (G_A and G_B) were initially activated by reducing with Na/K (molar ratio 1:3) alloy. Specifically, a drop of liquid Na/K alloy (molar ratio is 1:3) is dripped onto the surface of the sample and kept for 1.5 h (the reduction is saturated), resulting in the exclusive reduction of unprotected graphene. This new activation procedure by reducing graphene directly with the liquid Na/K alloy differs considerably from our previously developed method employing Na/K-DME (DME = dimehtoxyethane) solution. The use of neat Na/K alloy avoids the application of the PMMA-damaging DME solvent thus guaranteeing compatibility with the mask lithography process. Two different diazonium salts including 4-Bromobenzenediazonium-tetrafluoroborate and 4-Nitrobenzenediazonium-tetrafluoroborate were respectively dissolved in dried and degased ethanol (0.5mmol / mL). Then the liquid Na/K alloy can be simply blown away with inert Ar. As soon as removing of the Na/K alloy one drop of each diazonium salt solution was added immediately for 15 min. Afterwards, the reactions were terminated by removing reactants with ethanol. Subsequently, the two samples were exported from the glove-box and washed with additional 20 mL ethanol and 20 mL water. Then acetone was used to remove PMMA layer to give rise to the final G_A and G_B.

Raman spectroscopy and in particular, scanning-Raman spectroscopy- and microscopy (SRS and SRM) represents a very powerful monitoring tool for the investigation of covalent addend binding to graphene. This technique was therefore applied to characterize our 2D-patterned sheet architectures G_A and G_B. As shown in Fig. S3 and S4, with the increase of reduction time for G_A and G_B, the intensity of D-band increased progressively, suggesting that the degree of functionalization increases gradually. Finally, the average I_D/I_G ratio of G_A/G_B can be enhanced to ca. 2.6/2.8. Thus, it’s interesting that our method can control the degree of functionalization by manipulating the reduction time of graphene, which is of great importance for graphene chemistry.

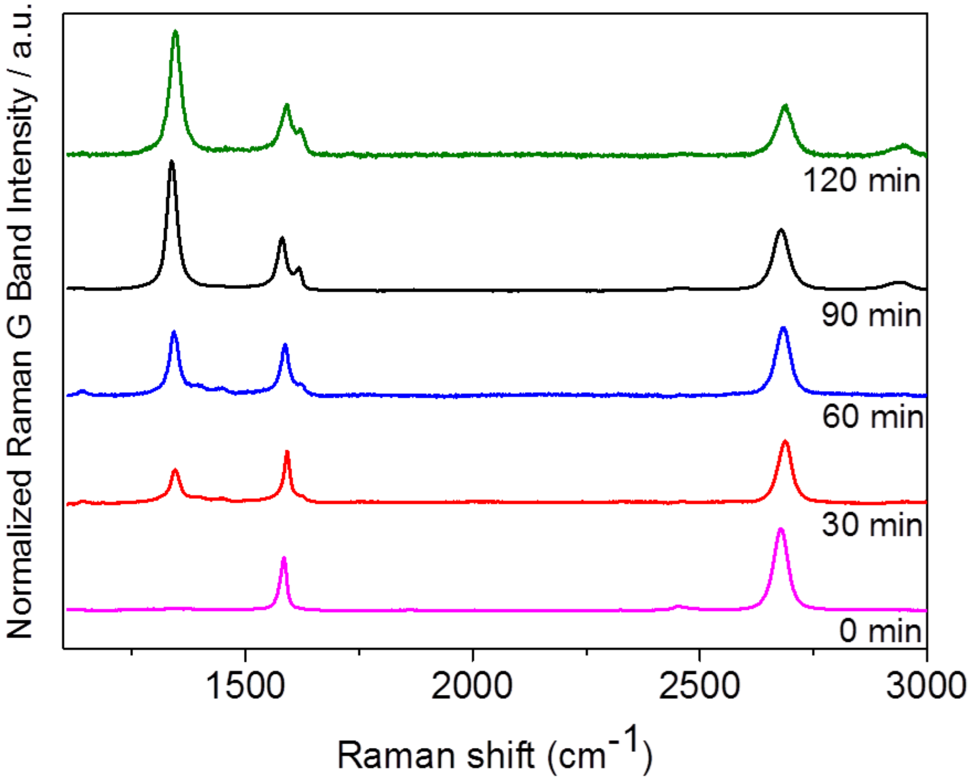

Figure S3. Raman spectra of patterning functionalizaition of graphene (G_A) upon different reduction time.

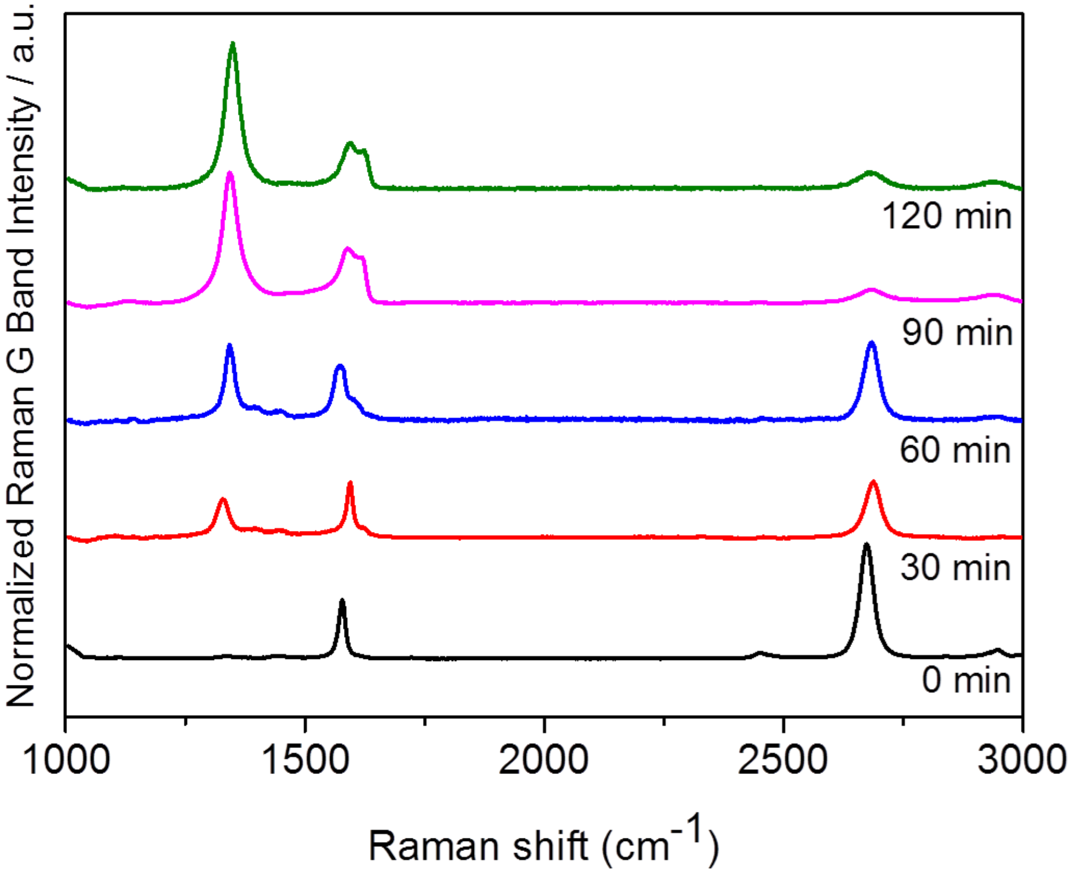

Figure S4. Raman spectra of patterning functionalization of graphene (G_B) upon different reduction time.

Table S1: Raman data of patterning functionalization of G_A and G_B upon different reduction time, λ_{exc} = 532 nm

Reduction Time / min	I_D/I_G for G_A	I_D/I_G for G_B
0	<0.1	<0.1
30	0.64	0.71
60	1.23	1.45
90	2.6	2.8
120	2.6	2.8

S3. ***Quantified local degree of functionalization of G_A and G_B.***

Previous studies have pointed out the correlation between I_D/I_G and mean defect distance L_D, with a maximum I_D/I_G ratio at a certain $L_{D\text{-crit}}$ value.[2, 3] In general, the $L_{D\text{-crit}}$ can be used as a boundary to distinguish the low density functionalization from the high density functionalization. To determine the L_D value under consideration, the width of the Raman bands is the core. As a consequence, the degree of functionalization of graphene can be quantified based on I_D/I_G ratio along with full width at half maxium (FWHM) of D peak. The determined I_D/I_G ratio combined with observed FWHM value (< 30 cm^{-1}) of D peak indicate the low density functionalization of G_A and the mean distance between defects (L_D) was calculated to be 6.2 nm. Following the quantified graphene functionalization method we introduced earlier,[4] the local degree of functionalization can be quantified to 0.060%. Based on the observed I_D/I_G ratio as well as FWHM of value (< 30 cm^{-1}) of D peak, the L_D was calculated to be 5.9 nm corresponding to the local degree of functionalization of 0.066% for G_B. Furthermore, the local degree of functionalization for both G_A and G_B under different annealing temperature has been quantified as well (Table S2). Besides, on basis of this method, we also quantified the degree of functionalization of previously reported patterning graphene functionalization for comparison (see Table S3).

Table S2: Quantified local degree of functionalization (θ) of G_A and G_B upon different annealing temperature.

θ \ °C	50	100	150	200	250	300	350	400
θ_{G_A} %	0.060	0.056	0.054	0.053	0.039	0.018	0.006	0.002
θ_{G_B} %	0.066	0.063	0.057	0.054	0.053	0.016	0.005	0.002

Table S3: Comparison of quantified degree of functionalization (θ) of previous reported cases (patterning graphene addition) and this work.

	Ref S5	Ref S6	Ref S7	Ref S8	This work
I_D / I_G	0.25	0.56	0.72	0.80	2.8 / 2.6
θ %	0.004	0.009	0.012	0.014	0.066 / 0.06

References:

[S1] J. C. Meyer, F. Eder, S. Kurasch, V. Skakalova, J. Kotakoski, H. J. Park, S. Roth, A. Chuvilin, S. Eyhusen, G. Benner, A. V. Krasheninnikov, U. Kaiser, *Phys. Rev. Lett.* **2012**, *108*, 196102-196108.

[S2] L. G. Cancado, A. jorio, E. H. Martins, F. F. Stavale, C. A. Achete, R. B. Capaz, M. V. O. Moutinho, A. Lombardo, T. S. Kulmala, A. C. Ferrari, *Nano Lett*, **2011**, *11*, 3190-3196.

[S3] M. M. Lucchese, F. Stavale, E. H. Martins Ferreira, C. Vilani, M. V. O. Moutinho, R. B. Capaz, C. A. Achete, A. Jorio, *Carbon*, **2010**, *48*, 1592-1597.

[S4] J. M. Englert, P. Vevera, K. C. Knirsch, R. A. Schäfer, F. Hauke, A. Hirsch, *ACSNano*, **2013**, *7*, 5472-5482.

[S5] J. Li, M. Li, L. L. Zhou, S. Y. Lang, H. Y. Lu, D. Wang, C. F. Chen, L. J. Wan, *J. Am. Chem. Soc.* **2016**, *138*, 7448-7451.

[S6] S. Bian, A. M. Scott, Y. Cao, Y. Liang, S. Osuna, K. N. Houk, A. B. Braunschweig, *J. Am. Chem. Soc.* **2013**, *135*, 9240-9243.

[S7] L. H. Liu, G. Zorn, D. G. Castner, R. Solanki, M. M. Lerner, M. Yan, *J. Mater. Chem.* **2010**, *20*, 5041-504.

[S8] Z. Z. Sun, C. L. Pint, D. C. Marcano, C. G. Zhang, J. Yao, G. D. Ruan, Z. Yan, Y. Zhu, R. H. Hauge, J. M. Tour, *Nature. Commun.* **2011**, *2*, 559-564.
